# Physics and proof theory

**DOI:** 10.1016/j.amc.2011.06.058

**Published:** 2012-09-15

**Authors:** Bruno Woltzenlogel Paleo

**Affiliations:** Institut für Computersprachen, Vienna University of Technology, Austria; INRIA Nancy, Equipe VeriDis, Bâtiment B 615, rue du Jardin Botanique F-54602 Villers-lés-Nancy, France

**Keywords:** Proof theory, Physics, Formalization of science

## Abstract

Axiomatization of Physics (and science in general) has many drawbacks that are correctly criticized by opposing philosophical views of science. This paper shows that, by giving formal proofs a more prominent role in the formalization, many of the drawbacks can be solved and many of the opposing views are naturally conciliated. Moreover, this approach allows, by means of proof theory, to open new conceptual bridges between the disciplines of Physics and Computer Science.

## 1 Introduction

“Science is built up with facts, as a house is with stones. But a collection of facts is no more a science than a heap of stones is a house.” – Poincaré

Foundational works on the formalization of Physics typically consider a physical theory as a collection of facts, i.e. as a set of sentences closed under logical consequence. However, not as much attention has been given to studying how these facts are or should be organized or, equivalently, how the physical theory is or should be structured. Usually, the only structure considered is a distinction of facts either as axioms or as derivable theorems (i.e. axiomatization). Although simple, this approach has a few drawbacks.

Firstly, from an epistemological point of view, the mentioned approach suffers from a logical omniscience problem: although physicists might know the axioms of their theories, it is certainly not the case that they know all the logical consequences of these axioms, simply because they have limited reasoning resources. Therefore, the approach of defining a theory as a set of sentences closed under logical consequence fails to capture the notion of theory as perceived by resource-bounded physicists; it is just an idealized approximation.

Secondly, the selection of which facts should be taken as axioms is arbitrary and frequently based on subjective criteria such as elegance. For example, there are axiomatizations of Physics that do not rely on the rather natural concepts of space and time [18]. Should they be considered more elegant, useful or correct?

Finally, there are cases of physical theories, such as Newtonian mechanics and Lagrangean mechanics, that are considered equivalent to each other according to the mentioned approach, because their sets of sentences closed under logical equivalence are the same, even though they actually differ significantly in how easily they can be used to solve certain classes of problems.

The second and third drawbacks mentioned above have been the main reasons for criticism on the whole enterprise of formalizing science [20]. However, they actually only apply to (unstructured) axiomatization. As a response to the criticism, there was a rise of semantic approaches, which adopted a more model-theoretic approach to the formalization of science [20]. Advances in the sibling discipline of proof theory, on the other hand, have not been given much attention.

The main goal of this paper is to advocate in favor of a more prominent role for proofs in the formalization of Physics, and consequently, for proof theory in approaches to Hilbert’s sixth problem [22] and in studies of the foundations of Physics. If a physical theory is considered not as a collection of sentences closed under logical consequence, but rather as a collection of proofs, the above mentioned drawbacks are naturally solved. Non-idealized resource-bounded physicists know only what they have proved so far. Axioms are simply the assumptions of the proofs contained in the physical theory. And various physical theories can be objectively compared with respect to the structure of the proofs they contain. This proposal is in line with current work in the formalization of mathematics, where mathematical knowledge is formalized as collections of proofs with the assistance of interactive theorem provers.^1^

The use of proofs to formalize computations of solutions of physical problems is exemplified with a simple problem of Newtonian mechanics in Section 3. The proof calculus used, known as sequent calculus, is briefly explained in Section 2. Finally, Section 4 discusses the benefits and challenges of using proofs in the formalization of Physics, from philosophical and computational points of view.

## 2 The sequent calculus **LK**^**P**^

The formal proofs in this paper are written in an extension of Gentzen’s sequent calculus **LK** [11]. A *sequent* is a pair *Γ* ⊢ *Δ*, where *Γ* (the antecedent) and *Δ* (the succedent) are multisets of formulas, with the intuitive intended meaning that the disjunction of the formulas in *Δ* is provable assuming the formulas in *Γ*. An **LK**-proof is a (hyper) tree of sequents, such that the leaves are *axiom sequents* of the form *F* ⊢ *F*, where *F* is an arbitrary formula, and the (hyper) edges are instances of the inference rules specified by the calculus. The sequent calculus **LK** has inference rules for propositional connectives (e.g. ∨, → , ¬ and ∧), as exemplified below for the ∧ connective:

$$\frac{\Gamma ⊢\Delta \text{,}A\;\Pi ⊢\Lambda \text{,}B}{\Gamma \text{,}\Pi ⊢\Delta \text{,}\Lambda \text{,}A\wedge B}\wedge :r\;\frac{A\text{,}\Gamma ⊢\Delta }{A\wedge B\text{,}\Gamma ⊢\Delta }\wedge :l1\;\frac{A\text{,}\Gamma ⊢\Delta }{B\wedge A\text{,}\Gamma ⊢\Delta }\wedge :l2\text{.}$$

The following inference rules for quantifiers are also available (with the important restriction that the ∀ : *r* and ∃ : *l* rules must satisfy the eigenvariable condition, i.e. the variable *α* must occur neither in *Γ* nor in *Δ* nor in *A*):

$$\begin{array}{cc} & \frac{A\{x\;\leftarrow \;t\}\text{,}\Gamma ⊢\Delta }{(\forall x)A\text{,}\Gamma ⊢\Delta }\forall :l\;\frac{\Gamma ⊢\Delta \text{,}A\{x\;\leftarrow \;\alpha \}}{\Gamma ⊢\Delta \text{,}(\forall x)A}\forall :r\text{,}\\ & \frac{A\{x\;\leftarrow \;\alpha \}\text{,}\Gamma ⊢\Delta }{(\exists x)A\text{,}\Gamma ⊢\Delta }\exists :l\;\frac{\Gamma ⊢\Delta \text{,}A\{x\;\leftarrow \;t\}}{\Gamma ⊢\Delta \text{,}(\exists x)A}\exists :r\text{.} \end{array}$$

Moreover, the sequent calculus **LK** also provides structural rules such as contraction (*c*_*l*_ and *c*_*r*_, needed to indicate when facts must be used more than once in a proof), weakening (*w*_*l*_ and *w*_*r*_, needed to indicate when facts are not really needed in the proof) and, most importantly, the cut rule, which, as discussed in Section 4, eases the structured formalization of Physics:

$$\frac{\Gamma ⊢\Delta \text{,}F\;F\text{,}\Gamma ⊢\Delta }{\Gamma ⊢\Delta }\;\mathrm{cut}\text{.}$$

However, the pure sequent calculus **LK** does not provide any built-in support for equality handling, arithmetical simplifications, and differentiation and integration. Therefore, formalizing Physics in the pure sequent calculus **LK** would be tedious and uncomfortable, since the lack of built-in support would require adding several additional assumptions to the antecedents of the sequents, which would render the proofs large, unreadable and difficult to construct. The sequent calculus **LK**^**P**^ addresses this issue by extending **LK** with the following rules:

• **Built-in Support for Equality:**
  $$\begin{array}{cc} & \frac{\Gamma \text{,}s=t\text{,}A[t]⊢\Delta }{\Gamma \text{,}s=t\text{,}A[s]⊢\Delta }\;{=}_{l}\;\frac{\Gamma \text{,}s=t⊢\Delta \text{,}A[t]}{\Gamma \text{,}s=t⊢\Delta \text{,}A[s]}\;{=}_{r}\text{,}\\ & \frac{\Gamma \text{,}s=t\text{,}A[s]⊢\Delta }{\Gamma \text{,}s=t\text{,}A[t]⊢\Delta }\;{=}_{l}\;\frac{\Gamma \text{,}s=t⊢\Delta \text{,}A[s]}{\Gamma \text{,}s=t⊢\Delta \text{,}A[t]}\;{=}_{r}\text{,} \end{array}$$
  where *s* and *t* do not contain variables that are bound in *A*.
• **Built-in Support for Definitions**^2^: They correspond directly to the *extension principle* and introduce new predicate and function symbols as abbreviations for formulas and terms. Let *A*[*x*_1_, … , *x*_*k*_] be an arbitrary formula with free-variables *x*_1_, … , *x*_*k*_ and *P* be a *new k*-ary predicate symbol defined by *P*(*x*_1_, … , *x*_*k*_) ↔ *A*[*x*_1_, … , *x*_*k*_]. Let *t*[*x*_1_, … , *x*_*k*_] be an arbitrary term with free-variables *x*_1_, … , *x*_*k*_ and *f* be a *new k*-ary function symbol defined by *f*(*x*_1_, … , *x*_*k*_) = *t*[*x*_1_, … , *x*_*k*_]. Then, for arbitrary sequences of terms *t*_1_, … , *t*_*k*_, the rules are:
  $$\begin{array}{cc} & \frac{A[t_{1}\text{,}\ldots \text{,}t_{k}]\text{,}\Gamma ⊢\Delta }{P(t_{1}\text{,}\ldots \text{,}t_{k})\text{,}\Gamma ⊢\Delta }\;d_{l}\;\frac{\Gamma ⊢\Delta \text{,}A[t_{1}\text{,}\ldots \text{,}t_{k}]}{\Gamma ⊢\Delta \text{,}P(t_{1}\text{,}\ldots \text{,}t_{k})}\;d_{r}\\ & \frac{F[t[t_{1}\text{,}\ldots \text{,}t_{k}]]\text{,}\Gamma ⊢\Delta }{F[f(t_{1}\text{,}\ldots \text{,}t_{k})]\text{,}\Gamma ⊢\Delta }\;d_{l}\;\frac{\Gamma ⊢\Delta \text{,}F[t[t_{1}\text{,}\ldots \text{,}t_{k}]]}{\Gamma ⊢\Delta \text{,}F[f(t_{1}\text{,}\ldots \text{,}t_{k})]}\;d_{r} \end{array}$$
• **Built-in Support for Simplification:** Let *t* (or *t*′) be obtainable from *t*′ (*t*) by algebraic or arithmetical simplifications.^3^ Then the rules are:
  $$\frac{F[t^{\prime }]\text{,}\Gamma ⊢\Delta }{F[t]\text{,}\Gamma ⊢\Delta }\;s_{l}\;\frac{\Gamma ⊢\Delta \text{,}F[t^{\prime }]}{\Gamma ⊢\Delta \text{,}F[t]}\;s_{r}$$
• **Built-in Support for Integration and Differentiation**^4^: Let *t*_1_(*t*_2_) be a term denoting the integral of the function denoted by $t_{1}^{\prime }(t_{2}^{\prime })$ on the interval (*x*_1_, *x*_2_). Then the rules are:
  $$\frac{F[t_{1}=t_{2}]\text{,}\Gamma ⊢\Delta }{F[t_{1}^{\prime }=t_{2}^{\prime }]\text{,}\Gamma ⊢\Delta }\;\int_{x_{1}}^{x_{2}}:l\;\frac{\Gamma ⊢\Delta \text{,}F[t_{1}^{\prime }=t_{2}^{\prime }]}{\Gamma ⊢\Delta \text{,}F[t_{1}=t_{2}]}\int_{x_{1}}^{x_{2}}:r$$

## 3 A simple example: energy conservation as a cut

To solve problems of Physics, certain invariants (such as energy) are frequently used. This is so because solving problems by using a derived principle (such as the principle of energy conservation) is usually easier than solving them by using the most basic physical laws or axioms. This section intends to exemplify how problem solution can generally be seen from a proof-theoretic perspective in which the use of derived principles corresponds to an implicit use of the cut rule. The following simple problem of Newtonian mechanics shall be considered.

An object of mass *m* is dropped from height *h*_0_ and with initial velocity equal to zero. The only force acting on the object is the force of gravity (with an intensity *mg*). What is the velocity of the object when its height is equal to zero?

A typical solution (Solution 1) to this problem uses the principle of energy conservation, as follows.

1. Let *t*_*f*_ be the time when the object reaches height zero.
2. According to the principle of energy conservation, *e*(*t*_*f*_) = *e*(0), i.e. the energy at *t*_*f*_ is equal to the initial energy.
3. Hence, by definition of gravitational potential energy in a uniform gravitational field and by definition of kinetic energy, $\mathrm{mgh}(t_{f})+m\frac{\overset{˙}{h}{\left(t_{f}\right)}^{2}}{2}=\mathrm{mgh}(0)+m\frac{\overset{˙}{h}{\left(0\right)}^{2}}{2}$.
4. According to the initial conditions, *h*(0) = *h*_0_ and $\overset{˙}{h}(0)=0$. Moreover, by assumption, *h*(*t*_*f*_) = 0. Therefore, $m\frac{\overset{˙}{h}{\left(t_{f}\right)}^{2}}{2}={\mathrm{mgh}}_{0}$.
5. Hence, the result is $\overset{˙}{h}(t_{f})=-\sqrt{2{\mathrm{gh}}_{0}}$.

Another solution (Solution 2) computes the velocity as a function of time by integrating the acceleration produced by the gravitational force. Then it determines the time when the object reaches height zero, and computes the velocity at that time. The details are shown below.

1. According to Newton’s second law of motion, $f(t)=m\overset{¨}{h}(t)$ at any time *t*. Moreover, the uniform gravitational field produces a force *f*(*t*) = −*mg*. Hence, $\overset{¨}{h}(t)=-g$.
2. By integration, $\overset{˙}{h}(t)=-\mathrm{gt}+\overset{˙}{h}(0)$.
3. According to the initial conditions, $\overset{˙}{h}(0)=0$, and hence $\overset{˙}{h}(t)=-\mathrm{gt}$.
4. By integration again, $h(t)=-g\frac{t^{2}}{2}+h(0)$.
5. According to the initial conditions, *h*(0) = *h*_0_, and hence $h(t)=-g\frac{t^{2}}{2}+h_{0}$.
6. For *h*(*t*_*f*_) = 0 to hold, it must be the case that $t_{f}=\sqrt{\frac{2h_{0}}{g}}$.
7. Hence $\overset{˙}{h}(t_{f})=-g\sqrt{\frac{2h_{0}}{g}}$, which can be simplified to $\overset{˙}{h}(t_{f})=-\sqrt{2{\mathrm{gh}}_{0}}$.

Solution 2 is simpler in the sense that it uses only the basic physical laws of motion (here assumed to be Newton’s laws of motion) and of uniform gravitational fields. Solution 1, on the other hand, assumes that energy is conserved, without actually proving it from Newton’s basic laws.

In order to view problem solving from a proof theoretic perspective, it is necessary to formalize problem solving as theorem proving, as is usual in logic programming. In the example above, the problem can be stated as the following theorem to be proved:

$$(\exists t^{\prime })(h(t^{\prime })=0\wedge (\exists v)\;\overset{˙}{h}(t^{\prime })=v)\text{.}$$

Solving the given problem then consists of finding a proof of the theorem above such that *v* is instantiated by a ground term. The solution is then contained in the proof, precisely in the instantiations used for the existentially quantified variables. Interestingly, formalizing the problem as a theorem to be proved enforces the explicit mention of the hidden assumption that the height eventually becomes zero; otherwise the variable *t*′ would be free and the theorem would be open.

Traditionally, works of axiomatization have formalized physical laws as axioms that are supposed to be used as assumptions in proofs [20]. In a more modern proof-theoretical approach, however, definition rules often provide a more convenient alternative. The axioms corresponding to certain physical laws can be seen as defining new symbols. This is the case, for example, of Newton’s second law, which states that force equals mass times acceleration $\left(f(t)=m\overset{¨}{h}(t)\right)$. It can be seen as defining the function symbol *f*. Similarly, the equation for energy of a single object in a uniform newtonian gravitational field $\left(e(t)=\mathrm{mgh}(t)+m\frac{\overset{˙}{h}{\left(t\right)}^{2}}{2}\right)$ can be seen as defining the function symbol *e*. For convenience, the defined predicate symbols below are also used in the following formal proofs:

**Table:** 

Initial conditions:	$I↔\mathrm{Init}\;↔\;h(0)=h_{0}\wedge \overset{˙}{h}(0)=0$
Uniform gravitation:	*G* ↔ *Gravity* ↔ (∀*t*)(*f*(*t*) = −*mg*)
Fall of the object:	*F* ↔ *Fall* ↔ (∃*t*) *h*(*t*) = 0
Energy conservation:	*EC* ↔ *EnergyConservation* ↔ (∀*t*_*i*_)(∀*t*_*j*_) *e*(*t*_*i*_) = *e*(*t*_*j*_)

Solution 1 can be easily formalized as the proof *φ*_1_ below (where $\varphi_{1}^{\prime }$ is a subproof consisting of the single axiom sequent *h*(*t*_*f*_) = 0 ⊢ *h*(*t*_*f*_) = 0):

$$\begin{array}{cc} & \overset{˙}{h}(t_{f})=-\sqrt{2{\mathrm{gh}}_{0}}⊢\overset{˙}{h}(t_{f})=-\sqrt{2{\mathrm{gh}}_{0}}\;\exists_{r}\\ & \overset{˙}{h}(t_{f})=-\sqrt{2{\mathrm{gh}}_{0}}⊢(\exists v)\;\overset{˙}{h}(t_{f})=v\;s_{l}\\ & \mathrm{mg}0+m\frac{\overset{˙}{h}{\left(t_{f}\right)}^{2}}{2}={\mathrm{mgh}}_{0}+m\frac{0^{2}}{2}⊢(\exists v)\overset{˙}{h}(t_{f})=v\;w_{l}\\ & h(t_{f})=0\text{,}h(0)=h_{0}\text{,}\overset{˙}{h}(0)=0\text{,}\mathrm{mg}0+m\frac{\overset{˙}{h}{\left(t_{f}\right)}^{2}}{2}={\mathrm{mgh}}_{0}+m\frac{0^{2}}{2}⊢(\exists v)\;\overset{˙}{h}(t_{f})=v\;{=}_{l}\\ & h(t_{f})=0\text{,}h(0)=h_{0}\text{,}\overset{˙}{h}(0)=0\text{,}\mathrm{mgh}(t_{f})+m\frac{\overset{˙}{h}{\left(t_{f}\right)}^{2}}{2}=\mathrm{mgh}(0)+m\frac{\overset{˙}{h}{\left(0\right)}^{2}}{2}⊢(\exists v)\;\overset{˙}{h}(t_{f})=v\;d_{l}\\ & h(t_{f})=0\text{,}h(0)=h_{0}\text{,}\overset{˙}{h}(0)=0\text{,}e(t_{f})=e(0)⊢(\exists v)\;\overset{˙}{h}(t_{f})=v\;\forall_{l}\\ & h(t_{f})=0\text{,}h(0)=h_{0}\text{,}\overset{˙}{h}(0)=0\text{,}(\forall t_{i})(\forall t_{j})\;e(t_{i})=e(t_{j})⊢(\exists v)\;\overset{˙}{h}(t_{f})=v\;\forall_{l}\\ & \varphi_{1}^{\prime }\;h(t_{f})=0\text{,}h(0)=h_{0}\text{,}\overset{˙}{h}(0)=0\text{,}(\forall t_{i})(\forall t_{j})\;e(t_{i})=e(t_{j})⊢(\exists v)\;\overset{˙}{h}(t_{f})=v\;\wedge_{r}\\ & h(t_{f})=0\text{,}h(t_{f})=0\text{,}h(0)=h_{0}\text{,}\overset{˙}{h}(0)=0\text{,}(\forall t_{i})(\forall t_{j})\;e(t_{i})=e(t_{j})⊢h(t_{f})=0\wedge (\exists v)\;\overset{˙}{h}(t_{f})=v\;c_{l}\\ & h(t_{f})=0\text{,}h(0)=h_{0}\text{,}\overset{˙}{h}(0)=0\text{,}(\forall t_{i})(\forall t_{j})\;e(t_{i})=e(t_{j})⊢h(t_{f})=0\wedge (\exists v)\;\overset{˙}{h}(t_{f})=v\;\exists_{r}\\ & h(t_{f})=0\text{,}h(0)=h_{0}\text{,}\overset{˙}{h}(0)=0\text{,}(\forall t_{i})(\forall t_{j})\;e(t_{i})=e(t_{j})⊢(\exists t^{\prime })(h(t^{\prime })=0\wedge (\exists v)\;\overset{˙}{h}(t^{\prime })=v)\;\exists_{l}\\ & (\exists t)\;h(t)=0\text{,}h(0)=h_{0}\text{,}\overset{˙}{h}(0)=0\text{,}(\forall t_{i})(\forall t_{j})\;e(t_{i})=e(t_{j})⊢(\exists t^{\prime })(h(t^{\prime })=0\wedge (\exists v)\;\overset{˙}{h}(t^{\prime })=v)\;\wedge_{l}\\ & (\exists t)\;h(t)=0\text{,}h(0)=h_{0}\wedge \overset{˙}{h}(0)=0\text{,}(\forall t_{i})(\forall t_{j})\;e(t_{i})=e(t_{j})⊢(\exists t^{\prime })(h(t^{\prime })=0\wedge (\exists v)\;\overset{˙}{h}(t^{\prime })=v)\;d_{l}\\ & \mathrm{Fall}\text{,}\mathrm{Init}\text{,}\mathrm{EnergyConservation}⊢(\exists t^{\prime })(h(t^{\prime })=0\wedge (\exists v)\;\overset{˙}{h}(t^{\prime })=v) \end{array}$$

Solution 2 can be formalized as the following proof *φ*_2_:

$$\begin{array}{cc} & \;\;\;\;\;\overset{˙}{h}\left(\sqrt{\frac{2h_{0}}{g}}\right)=-\sqrt{2{\mathrm{gh}}_{0}}⊢\overset{˙}{h}\left(\sqrt{\frac{2h_{0}}{g}}\right)=-\sqrt{2{\mathrm{gh}}_{0}}\;\exists_{r}\\ & \;\;\;\;\;\overset{˙}{h}\left(\sqrt{\frac{2h_{0}}{g}}\right)=-\sqrt{2{\mathrm{gh}}_{0}}⊢(\exists v)\overset{˙}{h}\left(\sqrt{\frac{2h_{0}}{g}}\right)=v\;s_{l}\\ & \;\;\;\;\;\overset{˙}{h}\left(\sqrt{\frac{2h_{0}}{g}}\right)=-g\sqrt{\frac{2h_{0}}{g}}⊢(\exists v)\overset{˙}{h}\left(\sqrt{\frac{2h_{0}}{g}}\right)=v\;\forall_{l}\\ & h\left(\sqrt{\frac{2h_{0}}{g}}\right)=0⊢h\left(\sqrt{\frac{2h_{0}}{g}}\right)=0\;w_{l}\;(\forall t)(\overset{˙}{h}(t)=-\mathrm{gt})⊢(\exists v)\;\overset{˙}{h}\left(\sqrt{\frac{2h_{0}}{g}}\right)=v\;w_{l}\\ & h(0)=h_{0}\text{,}h\left(\sqrt{\frac{2h_{0}}{g}}\right)=0⊢h\left(\sqrt{\frac{2h_{0}}{g}}\right)=0\;\overset{˙}{h}(0)=0\text{,}(\forall t)(\overset{˙}{h}(t)=-\mathrm{gt})⊢(\exists v)\overset{˙}{h}\left(\sqrt{\frac{2h_{0}}{g}}\right)=v\;\wedge_{r}\\ & h(0)=h_{0}\text{,}\overset{˙}{h}(0)=0\text{,}h\left(\sqrt{\frac{2h_{0}}{g}}\right)=0\text{,}(\forall t)(\overset{˙}{h}(t)=-\mathrm{gt})⊢h\left(\sqrt{\frac{2h_{0}}{g}}\right)=0\wedge (\exists v)\;\overset{˙}{h}\left(\sqrt{\frac{2h_{0}}{g}}\right)=v\;\exists_{r}\\ & h(0)=h_{0}\text{,}\overset{˙}{h}(0)=0\text{,}h\left(\sqrt{\frac{2h_{0}}{g}}\right)=0\text{,}(\forall t)(\overset{˙}{h}(t)=-\mathrm{gt})⊢(\exists t^{\prime })(h(t^{\prime })=0\wedge (\exists v)\;\overset{˙}{h}(t^{\prime })=v)\;s_{l}\\ & h(0)=h_{0}\text{,}\overset{˙}{h}(0)=0\text{,}h\left(\sqrt{\frac{2h_{0}}{g}}\right)=-g\frac{{\left(\sqrt{\frac{2h_{0}}{g}}\right)}^{2}}{2}+h_{0}\text{,}(\forall t)(\overset{˙}{h}(t)=-\mathrm{gt})⊢(\exists t^{\prime })(h(t^{\prime })=0\wedge (\exists v)\overset{˙}{h}(t^{\prime })=v)\;\forall_{l}\\ & h(0)=h_{0}\text{,}\overset{˙}{h}(0)=0\text{,}(\forall t)(h(t)=-g\frac{t^{2}}{2}+h_{0})\text{,}(\forall t)(\overset{˙}{h}(t)=-\mathrm{gt})⊢(\exists t^{\prime })(h(t^{\prime })=0\wedge (\exists v)\overset{˙}{h}(t^{\prime })=v)\;{=}_{l}\\ & h(0)=h_{0}\text{,}\overset{˙}{h}(0)=0\text{,}(\forall t)(h(t)=-g\frac{t^{2}}{2}+h(0))\text{,}(\forall t)(\overset{˙}{h}(t)=-\mathrm{gt})⊢(\exists t^{\prime })(h(t^{\prime })=0\wedge (\exists v)\;\overset{˙}{h}(t^{\prime })=v)\;\int_{l}\\ & h(0)=h_{0}\text{,}\overset{˙}{h}(0)=0\text{,}(\forall t)(\overset{˙}{h}(t)=-\mathrm{gt})\text{,}(\forall t)(\overset{˙}{h}(t)=-\mathrm{gt})⊢(\exists t^{\prime })(h(t^{\prime })=0\wedge (\exists v)\overset{˙}{h}(t^{\prime })=v)\;c_{l}\\ & h(0)=h_{0}\text{,}\overset{˙}{h}(0)=0\text{,}(\forall t)(\overset{˙}{h}(t)=-\mathrm{gt})⊢(\exists t^{\prime })(h(t^{\prime })=0\wedge (\exists v)\overset{˙}{h}(t^{\prime })=v)\;s_{l}\\ & h(0)=h_{0}\text{,}\overset{˙}{h}(0)=0\text{,}(\forall t)(\overset{˙}{h}(t)=-\mathrm{gt}+0)⊢(\exists t^{\prime })(h(t^{\prime })=0\wedge (\exists v)\overset{˙}{h}(t^{\prime })=v)\;{=}_{l}\\ & h(0)=h_{0}\text{,}\overset{˙}{h}(0)=0\text{,}(\forall t)(\overset{˙}{h}(t)=-\mathrm{gt}+\overset{˙}{h}(0))⊢(\exists t^{\prime })(h(t^{\prime })=0\wedge (\exists v)\;\overset{˙}{h}(t^{\prime })=v)\;\int_{l}\\ & h(0)=h_{0}\text{,}\overset{˙}{h}(0)=0\text{,}(\forall t)(\overset{¨}{h}(t)=-g)⊢(\exists t^{\prime })(h(t^{\prime })=0\wedge (\exists v)\;\overset{˙}{h}(t^{\prime })=v)\;s_{l}\\ & h(0)=h_{0}\text{,}\overset{˙}{h}(0)=0\text{,}(\forall t)(m\overset{¨}{h}(t)=-\mathrm{mg})⊢(\exists t^{\prime })(h(t^{\prime })=0\wedge (\exists v)\overset{˙}{h}(t^{\prime })=v)\;d_{l}\\ & h(0)=h_{0}\text{,}\overset{˙}{h}(0)=0\text{,}(\forall t)(f(t)=-\mathrm{mg})⊢(\exists t^{\prime })(h(t^{\prime })=0\wedge (\exists v)\;\overset{˙}{h}(t^{\prime })=v)\;\wedge_{l}\\ & h(0)=h_{0}\wedge \overset{˙}{h}(0)=0\text{,}(\forall t)(f(t)=-\mathrm{mg})⊢(\exists t^{\prime })(h(t^{\prime })=0\wedge (\exists v)\;\overset{˙}{h}(t^{\prime })=v)\;d_{l}\\ & \mathrm{Init}\text{,}\mathrm{Gravity}⊢(\exists t^{\prime })(h(t^{\prime })=0\wedge (\exists v)\overset{˙}{h}(t^{\prime })=v) \end{array}$$

As expected *φ*_1_ is not only smaller than *φ*_2_, but also simpler in the sense that it does not use integration. Furthermore, while in *φ*_2_ the time when the object hits the floor has to be computed explicitly (i.e. *t*′ is instantiated to a ground term), in *φ*_1_ this is not so (i.e. *t*′ is instantiated to a variable).

Solution 1 implicitly uses cuts, because *EnergyConservation* and *Fall* are not considered to be basic laws of Physics. In principle, *φ*_1_ must be composed with a proof *φ*_*E*_ of *EnergyConservation* and a proof *φ*_*F*_ of *Fall*. This is done with two cuts, as shown in the following proof *φ*:

$$\begin{array}{cc} & \;\;\varphi_{E}\;\;\;\varphi_{1}\\ & \varphi_{F}\;\mathrm{Gravity}⊢\mathrm{EC}\;\mathrm{Init}\text{,}\mathrm{Fall}\text{,}\mathrm{EC}⊢(\exists t^{\prime })(h(t^{\prime })=0\wedge (\exists v)\overset{˙}{h}(t^{\prime })=v)\;\mathrm{cut}\\ & \mathrm{Init}\text{,}\mathrm{Gravity}\;⊢\mathrm{Fall}\;\mathrm{Init}\text{,}\mathrm{Gravity}\text{,}\mathrm{Fall}⊢(\exists t^{\prime })(h(t^{\prime })=0\wedge (\exists v)\;\overset{˙}{h}(t^{\prime })=v)\;\mathrm{cut}\\ & \mathrm{Init}\text{,}\mathrm{Init}\text{,}\mathrm{Gravity}\text{,}\mathrm{Gravity}⊢(\exists t^{\prime })(h(t^{\prime })=0\wedge (\exists v)\;\overset{˙}{h}(t^{\prime })=v)\;c_{l}\\ & \mathrm{Init}\text{,}\mathrm{Gravity}⊢(\exists t^{\prime })(h(t^{\prime })=0\wedge (\exists v)\;\overset{˙}{h}(t^{\prime })=v) \end{array}$$

where *φ*_*F*_ is the proof below, proving that the object will eventually fall to height zero under the gravitational field and the initial conditions specified in the description of the problem:

$$\begin{array}{cc} & h\left(\sqrt{\frac{2h_{0}}{g}}\right)=0⊢h\left(\sqrt{\frac{2h_{0}}{g}}\right)=0\;\exists_{r}\\ & h\left(\sqrt{\frac{2h_{0}}{g}}\right)=0⊢(\exists t^{\prime })\;h(t^{\prime })=0\;s_{l}\\ & h\left(\sqrt{\frac{2h_{0}}{g}}\right)=-g\frac{{\left(\sqrt{\frac{2h_{0}}{g}}\right)}^{2}}{2}+h_{0}⊢(\exists t^{\prime })\;h(t^{\prime })=0\;\forall_{l}\\ & (\forall t)\left(h(t)=-g\frac{t^{2}}{2}+h_{0}\right)⊢(\exists t^{\prime })\;h(t^{\prime })=0\;w_{l}\\ & h(0)=h_{0}\text{,}(\forall t)(h(t)=-g\frac{t^{2}}{2}+h_{0})⊢(\exists t^{\prime })\;h(t^{\prime })=0\;{=}_{l}\\ & h(0)=h_{0}\text{,}(\forall t)\left(h(t)=-g\frac{t^{2}}{2}+h(0)\right)⊢(\exists t^{\prime })\;h(t^{\prime })=0\;\int_{l}\\ & h(0)=h_{0}\text{,}(\forall t)(\overset{˙}{h}(t)=-\mathrm{gt})⊢(\exists t^{\prime })\;h(t^{\prime })=0\;w_{l}\\ & h(0)=h_{0}\text{,}\overset{˙}{h}(0)=0\text{,}(\forall t)(\overset{˙}{h}(t)=-\mathrm{gt}+0)⊢(\exists t^{\prime })\;h(t^{\prime })=0\;{=}_{l}\\ & h(0)=h_{0}\text{,}\overset{˙}{h}(0)=0\text{,}(\forall t)(\overset{˙}{h}(t)=-\mathrm{gt}+\overset{˙}{h}(0))⊢(\exists t^{\prime })\;h(t^{\prime })=0\;\int_{l}\\ & h(0)=h_{0}\text{,}\overset{˙}{h}(0)=0\text{,}(\forall t)(\overset{¨}{h}(t)=-g)⊢(\exists t^{\prime })\;h(t^{\prime })=0\;s_{l}\\ & h(0)=h_{0}\text{,}\overset{˙}{h}(0)=0\text{,}(\forall t)(m\overset{¨}{h}(t)=-\mathrm{mg})⊢(\exists t^{\prime })\;h(t^{\prime })=0\;d_{l}\\ & h(0)=h_{0}\text{,}\overset{˙}{h}(0)=0\text{,}(\forall t)(f(t)=-\mathrm{mg})⊢(\exists t^{\prime })\;h(t^{\prime })=0\;\wedge_{l}\\ & h(0)=h_{0}\wedge \overset{˙}{h}(0)=0\text{,}(\forall t)(f(t)=-\mathrm{mg})⊢(\exists t^{\prime })\;h(t^{\prime })=0\;d\\ & \mathrm{Init}\text{,}\mathrm{Gravity}⊢\mathrm{Fall} \end{array}$$

And *φ*_*E*_ is the proof that energy is conserved in a uniform gravitational field:

$$\begin{array}{cc} & ⊢\mathrm{gh}(0)+\frac{\overset{˙}{h}{\left(0\right)}^{2}}{2}=\mathrm{gh}(0)+\frac{\overset{˙}{h}{\left(0\right)}^{2}}{2}\;w_{l}\\ & (\overset{˙}{h}(\alpha )=-g\alpha +\overset{˙}{h}(0))\text{,}(h(\alpha )=-g\frac{\alpha^{2}}{2}+\overset{˙}{h}(0)\alpha +h(0))\text{,}(\overset{˙}{h}(\beta )=-g\beta +\overset{˙}{h}(0))\text{,}(h(\beta )=-g\frac{\beta^{2}}{2}+\overset{˙}{h}(0)\beta +h(0))⊢\mathrm{gh}(0)+\frac{\overset{˙}{h}{\left(0\right)}^{2}}{2}=\mathrm{gh}(0)+\frac{\overset{˙}{h}{\left(0\right)}^{2}}{2}\;{=}_{r}^{*}\text{,}s_{r}^{*}\\ & (\overset{˙}{h}(\alpha )=-g\alpha +\overset{˙}{h}(0))\text{,}(h(\alpha )=-g\frac{\alpha^{2}}{2}+\overset{˙}{h}(0)\alpha +h(0))\text{,}(\overset{˙}{h}(\beta )=-g\beta +\overset{˙}{h}(0))\text{,}(h(\beta )=-g\frac{\beta^{2}}{2}+\overset{˙}{h}(0)\beta +h(0))⊢\mathrm{gh}(\alpha )+\frac{\overset{˙}{h}{\left(\alpha \right)}^{2}}{2}=\mathrm{gh}(\beta )+\frac{\overset{˙}{h}{\left(\beta \right)}^{2}}{2}\;\forall_{l}\\ & (\forall t)(\overset{˙}{h}(t)=-\mathrm{gt}+\overset{˙}{h}(0))\text{,}(\forall t)(h(t)=-g\frac{t^{2}}{2}+\overset{˙}{h}(0)t+h(0))\text{,}(\forall t)(\overset{˙}{h}(t)=-\mathrm{gt}+\overset{˙}{h}(0))\text{,}(\forall t)(h(t)=-g\frac{t^{2}}{2}+\overset{˙}{h}(0)t+h(0))⊢\mathrm{gh}(\alpha )+\frac{\overset{˙}{h}{\left(\alpha \right)}^{2}}{2}=\mathrm{gh}(\beta )+\frac{\overset{˙}{h}{\left(\beta \right)}^{2}}{2}\;c_{l}\\ & (\forall t)(\overset{˙}{h}(t)=-\mathrm{gt}+\overset{˙}{h}(0))\text{,}(\forall t)(h(t)=-g\frac{t^{2}}{2}+\overset{˙}{h}(0)t+h(0))⊢\mathrm{gh}(\alpha )+\frac{\overset{˙}{h}{\left(\alpha \right)}^{2}}{2}=\mathrm{gh}(\beta )+\frac{\overset{˙}{h}{\left(\beta \right)}^{2}}{2}\;\int_{l}\\ & (\forall t)(\overset{˙}{h}(t)=-\mathrm{gt}+\overset{˙}{h}(0))\text{,}(\forall t)(\overset{˙}{h}(t)=-\mathrm{gt}+\overset{˙}{h}(0))⊢\mathrm{gh}(\alpha )+\frac{\overset{˙}{h}{\left(\alpha \right)}^{2}}{2}=\mathrm{gh}(\beta )+\frac{\overset{˙}{h}{\left(\beta \right)}^{2}}{2}\;c_{l}\\ & (\forall t)(\overset{˙}{h}(t)=-\mathrm{gt}+\overset{˙}{h}(0))⊢\mathrm{gh}(\alpha )+\frac{\overset{˙}{h}{\left(\alpha \right)}^{2}}{2}=\mathrm{gh}(\beta )+\frac{\overset{˙}{h}{\left(\beta \right)}^{2}}{2}\;\int_{l}\\ & (\forall t)(\overset{¨}{h}(t)=-g)⊢\mathrm{gh}(\alpha )+\frac{\overset{˙}{h}{\left(\alpha \right)}^{2}}{2}=\mathrm{gh}(\beta )+\frac{\overset{˙}{h}{\left(\beta \right)}^{2}}{2}\;s\\ & (\forall t)(m\overset{¨}{h}(t)=-\mathrm{mg})⊢\mathrm{mgh}(\alpha )+m\frac{\overset{˙}{h}{\left(\alpha \right)}^{2}}{2}=\mathrm{mgh}(\beta )+m\frac{\overset{˙}{h}{\left(\beta \right)}^{2}}{2}\;d_{r}\\ & (\forall t)(m\overset{¨}{h}(t)=-\mathrm{mg})⊢e(\alpha )=e(\beta )\;d_{l}\\ & (\forall t)(f(t)=-\mathrm{mg})⊢e(\alpha )=e(\beta )\;\forall_{r}\\ & (\forall t)(f(t)=-\mathrm{mg})⊢(\forall t_{i})(\forall t_{j})\;e(t_{i})=e(t_{j})\;d\\ & \mathrm{Gravity}⊢\mathrm{EnergyConservation} \end{array}$$

## 4 Benefits and challenges of a proof-theoretical approach to the formalization of Physics

The following subsections are devoted to discussing what proof theory has to offer to the formalization of Physics, with emphasis on computational and philosophical aspects.

### 4.1 Cut-introduction

The example discussed in the previous section illustrates that an essential task of theoretical science is to invent or discover important concepts that are useful to solve problems, such as the principle of energy conservation in newtonian mechanics. Nevertheless, in a traditional axiomatization approach, such principles have no prominent role, because they are merely theorems derivable from the axioms. In a more proof-theoretic approach, on the other hand, proofs allow a structured formalization of the scientific knowledge, where important principles like energy conservation appear prominently formalized as active formulas in cut inferences, as shown in the formal proof *φ* of Section 3. Indeed, reductionism in science can generally be captured by the proof-theoretical notion of cut. Consequently, a significant part of the usual scientific activity can be formally described as cut-introduction.

Cut-introduction also leads to the compression of proofs. Although the general problem of finding the shortest proofs by means of cut-introduction is undecidable [5], there are a few preliminary algorithms that introduce simple cuts [10,15,24], and it has been shown that some techniques of machine learning, such as decision tree learning, can be seen as cut-introduction techniques from a proof-theoretical point of view [23]. Therefore, a potential benefit of using proofs to formalize Physics is the possibility of applying cut-introduction techniques in order to automatically discover useful physical concepts. However, it must be noted that current cut-introduction techniques are still not sophisticated enough to be applied to formalized proofs of Physics.

### 4.2 Cut-elimination

The problem of eliminating cuts from proofs is much easier than the problem of introducing cuts and has been much more deeply investigated [11,4]. By using cut-elimination algorithms, it might be possible to automatically transform a solution that uses a derived principle (i.e. a cut) such as energy conservation (e.g. Solution 1 in Section 3) into a solution that uses only the basic laws of a theory (e.g. Solution 2 in Section 3). This is advantageous in certain cases, for in a cut-free proof it is easy, via Gentzen’s Midsequent Theorem [11] or more general Herbrand sequent extraction algorithms [16], to extract a Herbrand disjunction [12] that contains instances of the quantified variables of the problem. For example, in the cut-free proof of Solution 2, the existentially quantified variable for the time when the object reaches height zero is instantiated by a ground term that denotes exactly when this happens. In the proof with cuts that formalizes Solution 1, on the other hand, it is instantiated by an eigenvariable, and hence the time when the object reaches height zero is not known. Therefore, cut-elimination could in principle be used as an algorithm that instantiates the variables of a problem that were left unsolved. However, even though this idea has been successfully used in mathematics [14], the challenge in the case of Physics is to make cut-elimination algorithms work with high-level calculi such as **LK**^**P**^.

### 4.3 Logic programming

The idea of formalizing a problem as a theorem and in such a way that its solution is in the instances used for the quantified variables in the proof is the fundamental principle behind the logic programming paradigm of computation, of which Prolog [19] is the most prominent language. Therefore, the proof-theoretical approach to the formalization of Physics brings a new paradigm of computation that might be the subject of studies from the point of view of Physics itself, as imperative computation, which is modeled by Turing machines, has been.

### 4.4 Functional programming and the Curry–Howard isomorphism

The Curry-Howard isomorphism [8] states that there is a correspondence between proofs of the implicational fragment of intuitionistic logic and lambda terms. A proof is essentially a functional program. Cut-elimination corresponds to beta-reduction, which is the execution of the program. Cut-introduction corresponds to structuring of the program and possibly to code reuse. By extrapolating this isomorphism, theories of Physics formalized as collections of proofs can be seen as collections of programs. This kind of computation, which is implicit in the formalization of Physics, is yet another link between Physics and computation that might be the target of future work.

### 4.5 Instrumentalism: truth versus usefulness

From an instrumentalistic viewpoint, “the most important function of a theory is not to organize or assert statements that are true or false but to furnish material principles of inference that may be used in inferring one set of facts from another”. This idea is supported by the proof-theoretical approach described here, as shown in the formal proof *φ*_2_ in Section 3, where Newton’s law of motion was not merely a statement; it was used as a principle of inference, in the form of a definition inference rule. Instrumentalism also judges theories by how useful they are in solving problems. The proof-theoretical approach naturally embraces this criterium of usefulness, since solutions to problems can be formalized as proofs, as shown by *φ*_1_ and *φ*_2_. And as the commitment to truth is not given up, it conciliates two opposing positions in the philosophy of science.

### 4.6 The evolution of theories

Another philosophical viewpoint that opposes axiomatization is that of Welt-anschauungen analyses, according to which science ought to be viewed as “an ongoing social enterprise [and] epistemic understanding of scientific theories could only be had by seeing the dynamics of theory development” [20]. From this perspective, a proper answer to the question ‘what is a scientific theory?’ cannot be formulated using only concepts such as axiomatization and semantics. It must take pragmatics into account and it must explicitly consider how the producers and consumers of a theory use and change it [21]. Proof theory conciliates formalization with this philosophical viewpoint in the following way: by defining scientific theories as collections of proofs, they can evolve by the addition of new proofs, and Kuhn’s major paradigm shifts can be seen as major proof transformations (e.g. cut-elimination, cut-introduction and addition of new definitions).

### 4.7 Algorithmic information theory

Algorithmic Information Theory (AIT) sees scientific theories as data compressed in the form of programs. It provides a very simple, elegant and general criterium to judge and compare theories: the smaller the program, the better the theory. However, the proponents of AIT are currently making an unfortunate choice of how to encode their data, and this causes the limitations of their approach. Diagrams in [7] suggest that theories/programs should correspond to axioms, and the execution of the program by a computer, regarded as an automated theorem prover, should output empirical data in the form of theorems. Therefore, they essentially adhere to the traditional Hilbert-style axiomatization approach, and hence they suffer the same drawbacks, which are nicely explained from a computational point of view in [7]. Two of them can be summarized as follows: in current AIT, computation time is ignored, because only program size matters; and the theory/program’s language is static, implying that new concepts can never emerge and the theory can never evolve.

Fortunately, proof theory can rescue AIT as well, and even provide further insight. The idea is that AIT’s principle of program-size minimality should be applied not to axioms (artificially encoded as programs) but rather to the proofs that formalize a scientific theory. From a conceptual point of view, it is clear that proof theory and AIT fit perfectly together, because proofs are already programs according to the (extrapolated) Curry-Howard isomorphism. The computation time that was previously ignored now appears explicitly as the length of proofs [17] and theories can naturally evolve by the addition and transformation of proofs in the collection, with new concepts emerging by the introduction of cuts and definition inferences.

Another indication that AIT and proof theory fit well together is the natural relation between cut-introduction and Kolmogorov complexity [13]. The Kolmogorov complexity *C*(*ψ*) of a proof *ψ* can be defined as the size of the shortest proof *ψ*′ that can be obtained by cut-introduction from *ψ* (and, conversely, such that *ψ* can be reconstructed from *ψ*′ by cut-elimination).

## 5 Conclusions

“It is unheard of to find a substantive example of a theory actually worked out as a logical calculus in the writings of most philosophers of science. Much handwaving is indulged in to demonstrate that this […] is simple in principle and only a matter of tedious detail, but concrete evidence is seldom given.” [21]. In Section 3, an example of problem solution in Newtonian mechanics has been successfully worked out in a sequent calculus extended with sophisticated simplification, integration and definition rules, inspired by recent advances in proof theory. These extensions are the key to the small size and significantly reduced amount of tedious detail in the obtained formal proofs.

Section 4 showed that this proof-theoretical approach successfully conciliates and unifies various philosophical views of science, such as formalism, instrumentalism and Weltanschauungen analyses. The essence of these achievements lies in seeing scientific theories not just as collections of facts, as assumed by traditional axiomatization. Scientific theories ought to be formalized as collections of proofs. The structure of scientific knowledge can be nicely formalized with cuts, and much of the scientific activity can be formally described as proof generation or proof transformation. The task of organizing knowledge, for example, can be formally described as cut-introduction.

Moreover, cut-introduction potentially compresses proofs, which can also be seen as programs according to the (extrapolated) Curry–Howard isomorphism. This indicates a tight relation between cut-introduction and Kolmogorov complexity, and thus the use of proofs clarifies, conceptually improves and solves some limitations of the ideas of algorithmic information theory with respect to the formalization of science.

The proof-theoretical approach advocated here should be seen not as competing against existing axiomatic and semantical approaches, but rather as complementing them by enriching their formalizations with structure.

Future work should concentrate on applying these proof-theoretical ideas to complement the formalization of more interesting physical theories, such as Relativity (e.g. [2]) and Quantum Mechanics (e.g. [1]); on improving proof assistants and proof-theoretical techniques, such as cut-elimination and cut-introduction, in order to support logical calculi at least as sophisticated as **LK**^**P**^; and on investigating the new links between Physics and Computation that are opened by proof theory.

## Footnotes

1 Examples of proof assistants are Mizar (http://mizar.uwb.edu.pl/), Coq (http://coq.inria.fr/) and Isabelle (http://www.cl.cam.ac.uk/research/hvg/Isabelle/).

2 Definition rules have been successfully used for formalization and analysis of mathematical proofs [3]. They are closely related to *superdeduction rules*[6], which can provide even more concise, natural and readable formal proofs. However, they are not as simple to describe, and hence definition rules have been used in this paper.

3 It is beyond the scope of this paper to define precisely the allowed simplifications. This kind of rule is inspired by *deduction modulo*, whose precise definitions can be found in [9]. In principle, simplification rules are not necessary, because they can be simulated by equality rules together with the arithmetical and algebraic axioms as additional assumptions in the antecedents of the sequents. However, the goal of simplification rules (and deduction modulo) is to hide uninteresting computational details of the underlying theories (e.g. arithmetics), in order to obtain concise formal proofs that show only interesting information related to the theory under investigation (e.g. newtonian mechanics).

4 Integration and Differentiation Rules have been inspired by emerging idea of integrating computer algebra systems and automated theorem provers.
